# Dependence of the sliding distance of a one-dimensional atom chain on initial velocity

**DOI:** 10.1038/s41598-017-16506-y

**Published:** 2017-11-24

**Authors:** Jian-Wen Li, Tong-Biao Wang, Nian-Hua Liu, Tianbao Yu

**Affiliations:** 1 School of Materials Science and Engineering, Nanchang University, Nanchang, 330031 China; 2 Department of physics, Nanchang University, Nanchang, 330031 China; 3 Institute for Advanced Study, Nanchang University, Nanchang, 330031 China; 4 Department of Science, Nanchang Institute of technology, Nanchang, 330099 China

## Abstract

In our daily lives, a body with a high initial velocity sliding freely on a rough surface moves a longer distance than that with a low initial velocity. However, such a phenomenon may not occur in the microscopic world. The dynamical behavior of a one-dimensional atom chain (1DAC) sliding on a substrate is investigated in this study by using a modified Frenkel–Kontorova model, in which the vibration of atoms on the substrate is considered. The dependence of sliding distance on initial velocity is examined. Result shows that although sliding distance is proportional to the initial value for most velocities, such a linear relation does not exist in some special velocities. This phenomenon is explained by a theoretical analysis of phonon excitation. The physical process is divided into three stages. The first stage is a superlubric sliding process with small amplitude of the vibrication of the atoms. The single-mode phonon is excited in the second stage. In the third stage, the system exhibits instability because of multiple-mode phonon excitations. In addition, the dependence of the coupling strength between 1DAC and the substrate is investigated. The findings are helpful in understanding the energy dissipation mechanism of friction.

## Introduction

Friction at the micro-/nano-scale has elicited much attention in the past two decades because of its important influence on the fabrication of micro-/nano-electromechanical systems^1–4^. The energy dissipation process accompanied by friction is highly complex, and understanding such a process is cumbersome. Therefore, various mechanisms have been proposed to study energy dissipation in friction^5–11^, and several models have been developed to investigate the physical mechanism of friction at the microscopic scale^12–19^. The mechanism of energy dissipation differs in different systems. For example, Sokoloff *et al*. proposed that the non-equilibrium phonons created during sliding are the major mechanism of energy dissipation^7–9^. Ciraci *et al*. studied energy dissipation when a nanoparticle (asperity) is connected to a surface of a sample sliding over the surface of another sample^11–14^. A microscopic analysis of the transient properties of energy dissipation via phonon discharge toward the substrate was presented^14^.

Aside from theoretical studies, several experimental investigations have also shown that phonons play important roles in energy dissipation^15,16^. For example, friction and dissipation in single and bilayer graphene films grown on SiC are different because of their significant difference in electron–phonon coupling; this difference can be observed through angle-resolved photoemission spectroscopy^15^. Given the fact that friction depends on the vibrational properties of surfaces, experimental studies have shown that the friction between hydrogen-terminated single-crystal diamond and silicon are higher than that between deuterium-terminated surfaces and silicon^16^. Several other factors can also lead to energy dissipation in the sliding process^20,21^. Excitation of electron–hole pairs is also a mechanism of frictional energy dissipation^17,18,22^. Such a dissipation channel has been demonstrated experimentally in the contact between two solids^23^.

In addition to the frictions between tips and substrates, frictions between surfaces have also been studied^24–27^. Many experiments have demonstrated that superlubricity exists between graphite microflakes. The self-retracting motion of graphite microflakes creates gigahertz-level nanoelectromechanical systems^25^. However, generating a stable oscillation is difficult because of the rapid loss of the kinetic energy of the microflakes in the self-retracting process. Although several mechanisms have been proposed to investigate energy dissipation in sliding friction, to the best of the authors’ knowledge, the mechanism of energy dissipation of sliding microflakes remains ambiguous. In particular, the dependence of the sliding distance of a microflake on its initial velocity has not been studied quantitatively. The current study used a modified Frenkel–Kontorova (FK) model and demonstrated that the sliding distance of a 1D atom chain (1DAC) is mainly determined by its initial velocity. When the initial velocity of a 1DAC is at a special value, although this value is very large, the 1DAC cannot move far. The physical mechanism of such a strange phenomenon was investigated in detail. The results showed that when the initial velocity of 1DAC is at a special value, the kinetic energy of 1DAC dissipates because of phonon excitations, which lead to a short sliding distance of 1DAC. All other initial velocities lead to superlubric sliding of 1DAC. In addition, the coupling strength between 1DAC and the substrate and the size of 1DAC exert a significant influence on the relationship between sliding distance and initial velocity.

## Model and Methods

**Figure 1:**
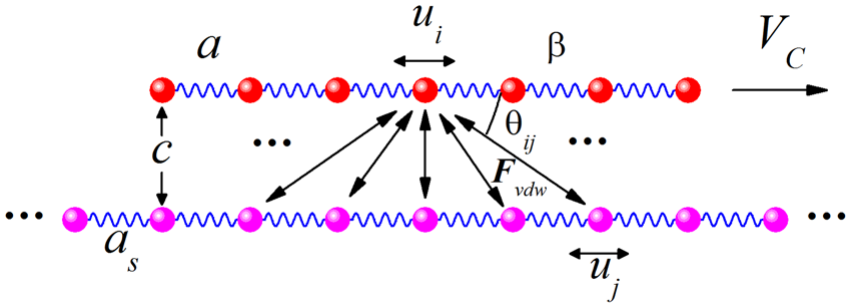
Schematic of the modified FK model.

The modified FK model shown in Fig. 1 was used to study the dynamical behavior of a 1DAC sliding against a substrate. The finite 1DAC of *N* atoms and the substrate with periodically arranged atoms were assumed to be made of the same type of atom. The interaction between the atom in the 1DAC and the atom in the substrate was described with the van der Waals (vdW) potential, and the interaction among the nearest-neighbor atoms in the same train was described by a harmonic force with spring stiffness *β* which is a constant determined by bond energy between the nearest neighbor atoms for the given 1DAC. The displayed simple model is suitable for the qualitative description of the physical process of sliding. Compared with the standard FK model in which the substrate is regarded as a fixed periodic potential^28–30^, the modified FK model used in this study assumes that the substrate atoms are vibrating with time. The atoms in the chain obey the following equation of motion.

1 $$m\frac{{d}^{2}{u}_{i}}{d{t}^{2}}=\beta ({u}_{i+1}-\,{u}_{i}-a)-\beta ({u}_{i}-\,{u}_{i-1}-a)-{\sum }_{j}\frac{d{U}_{ij}}{d{r}_{ij}}\cos \,{\theta }_{ij},$$

where *u*_*i*_ is the displacement of the *i*th atom in the atomic chain, *m* is the chain atom mass, *a* is the lattice constant, and *r*_*ij*_ is the distance between atom *i* in the chain and atom *j* in the substrate. ${\theta }_{ij}$ denotes the angle between the direction that atom *i* points to atom *j* and the sliding direction of the 1DAC.

The vdW potential between the atoms in the chain and the substrate is of the Lennard–Jones (LJ) type as follows^31^:

2 $${U}_{ij}=4\varepsilon [{(\frac{\sigma }{{r}_{ij}})}^{12}-{(\frac{\sigma }{{r}_{ij}})}^{6}].$$

The parameters of the LJ potential used in this study were $\varepsilon =2.39$ meV, $\sigma =0.341$ nm (diameter of the carbon atom). We calculate the interaction energies between 1DAC and substrate with help of Eq. (2) under different distance *c*, and find that the interaction energy is the lowest when *c* = 0.3626. The cutoff distance of the potential was set to $8\sigma$. A parameter was defined to characterize coupling intensity $\chi =\sqrt{24\varepsilon /m}/\sigma$, which has the same dimension as frequency. Thus, Eq. (1) is reduced to

3 $$\frac{{d}^{2}{u}_{i}}{d{t}^{2}}={\omega }_{s0}^{2}(u{}_{i+1}\,+\,{u}_{i-1}-2{u}_{i})+\sum _{j}{\chi }^{2}\sigma [2{(\frac{\sigma }{{r}_{ij}})}^{13}-{(\frac{\sigma }{{r}_{ij}})}^{7}]\cos \,{\theta }_{ij}.$$

Considering the substrate’s vibration, which can lead to phonon excitation, is practical because if two bodies sliding relative to each other have the same spring stiffness, then they have the same eigenfrequency. As a result, phonon excitation easily occurs. We assumed that the substrate vibrates parallel to the sliding direction and that the displacement of atom *j* follows ${u}_{s0}\,\cos \,[kj{a}_{s}-{\omega }_{s}(k)t]$. ${u}_{s0}$ is the vibrational amplitude, ${\omega }_{s}(k)$ is the frequency of phonon, its value can be determined by the dispersion relation of the phonon modes

4 $${\omega }_{s}(k)=2{\omega }_{s0}|\sin (ak/2)|,$$

where ${\omega }_{s0}=\sqrt{\beta /m}$ and *k* is the wave vector. Thus, the position of the *j*th atom on the substrate can be written as

5 $${u}_{j}=j{a}_{s}+{u}_{s0}\,\cos \,[kj{a}_{s}-{\omega }_{s}(k)t],$$

where *a*_*s*_ is the lattice constant of the substrate. The value of vibrational amplitude ${u}_{s0}$ can be estimated from the ordinary thermal motion of the atom. The average value of the kinetic energy of a classical harmonic oscillator within one period is

6 $$ < {E} > =\frac{1}{2}m{\omega }_{s0}^{2} < {u}_{s}^{2} > =\frac{1}{4}m{\omega }_{s0}^{2}{u}_{s0}^{2}.$$

According to the theorem of energy equipartition, ${u}_{s0}$ can be written as ${u}_{s0}=\sqrt{2{k}_{B}T/\beta }$, where *k*_*B*_ is the Boltzmann constant and *T* is temperature. On the basic of the phonon dispersion relation and the range of phonon energy of graphene in refs^32,33^, the value of ${\omega }_{s0}\approx 180$ THz can be evaluated. Furthermore, we can obtain the value of $\beta ={\omega }_{s0}^{2}m$. Therefore, *u*_*s*0_ has a magnitude of about 10^−3^ nm at room temperature. We set *u*_*s*0_ to 0.1*a* in the following calculations.

Prior to the calculation of dynamics of the sliding of the 1DAC, we considered its eigenmodes. In our numerical calculations, time, length and velocity were normalized with ${t}_{0}=h/\varepsilon$, ${a}_{s}=a$ and ${v}_{0}={a}_{s}/{t}_{0}$ respectively, where *h* is the Planck constant. Under free boundary conditions at the two ends, the motion equation of an arbitrary atom in the 1DAC is

7 $$\frac{{d}^{2}{u}_{i}}{d{t}^{2}}={\omega }_{{\rm{s}}0}^{2}({u}_{i+1}+\,{u}_{i-1}-\,2u{}_{i}).$$

Supposing that ${u}_{i}\propto \exp (-i\omega t)$, the displacement between neighbor atoms can be connected by the transmission matrix

8 $$(\begin{array}{c}{u}_{i+1}\\ {u}_{i}\end{array})=M(\begin{array}{c}{u}_{i}\\ {u}_{i-1}\end{array})=(\begin{array}{cc}2-\xi  & -1\\ 1 & 0\end{array})(\begin{array}{c}{u}_{i}\\ {u}_{i-1}\end{array}),$$

where $\xi ={\omega }^{2}/{\omega }_{{\rm{s}}0}^{2}$ and *ω* is the eigenfrequency. For a chain with *N* + 1 atoms, the atoms at two ends can also be connected by the matrix equation

9 $$(\begin{array}{c}{u}_{N}\\ {u}_{N-1}\end{array})={M}^{N-1}(\begin{array}{c}{u}_{1}\\ {u}_{0}\end{array}).$$

**Figure 2:**
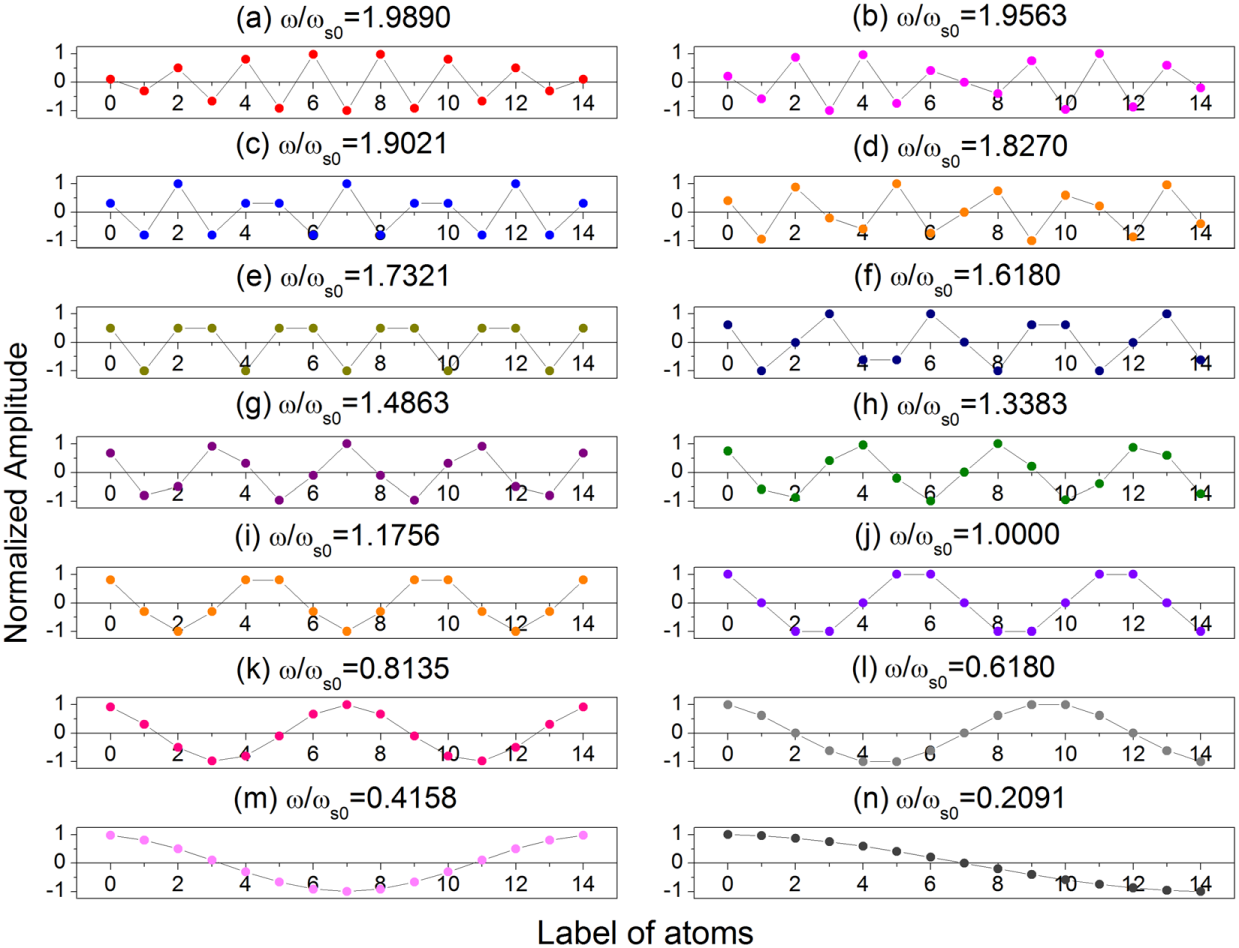
Displacement of atoms in 1DAC with *N* = 15 for all eigenfrequencies.

The *N*th power of matrix *M* was calculated with Chebyshev’s identities. Furthermore, considering the free boundary conditions at both ends, the eigenvalue equation was obtained as

10 $$({\xi }^{2}-4\xi +3)\sin (N-1)\alpha +(\xi -2)\sin (N-2)\alpha =0,$$

where *α* satisfies $\cos \,\alpha =1-\xi /2$. Thereafter, the amplitude of the eigenmodes for a given $\xi$ was obtained. The eigenfrequencies of 1DAC with 15 atoms were obtained by solving Eq. (10) numerically. The displacements of atoms in the 1DAC for different $\omega /{\omega }_{{\rm{s0}}}$ are shown in Fig. 2.

## Numerical Results and Discussion

A high initial velocity results in a large displacement of a macroscopic body in a free sliding process. However, by calculating the sliding of the 1DAC, we found that this condition is not always applicable for a nano-sized body.

**Figure 3:**
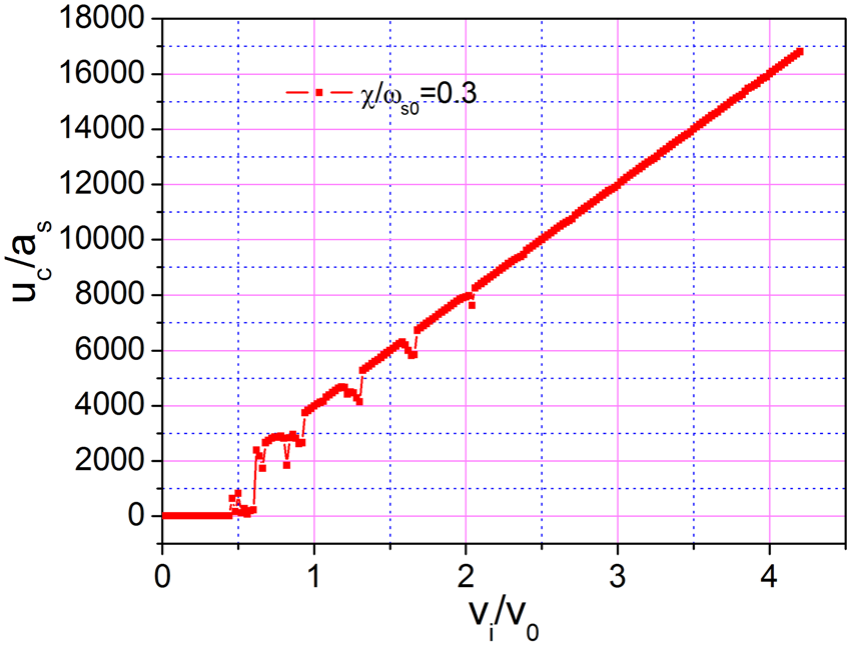
Largest displacement of center of mass as a function of initial velocity for *N* = 15, $k{a}_{{\rm{s}}}=0.15\pi$, and $\chi /{\omega }_{s0}=0.3$ in the time range of 0–4000.

To simulate the dynamical evolution of 1DAC, Eq. (3) was solved numerically with the fourth-order Runge–Kutta algorithm. We assumed that each atom of 1DAC is at its equilibrium position initially which is indicated in Table 1 for *N* = 15. Thus, we can obtain that the initial CM of 1DAC is ${u}_{c}/{a}_{s}=7.5$. Furthermore, the positions of atoms relative to CM can be obtained. All of the atoms move at the same velocity of *v*_*i*_ initially. Hence, the velocity of the center of mass (CM) is ${v}_{c}={v}_{i}$, and the elastic potential energy is zero. The displacement ${u}_{c}$ of CM as a function of initial velocity after the time range of $t/{t}_{0}=4000$ is shown in Fig. 3 for coupling strength $\chi /{\omega }_{s0}=0.3$. If the velocity of CM does not decrease, then CM displacement satisfies the linear relation ${u}_{c}={v}_{i}t$; this corresponds to the case in which the 1DAC moves in a superlubric manner without kinetic energy dissipation. If ${u}_{c} < {v}_{i}t$, then the velocity of CM decreases, and the kinetic energy is dissipated. Two cases emerge. One is that the displacement satisfies the linear relation ${u}_{c}={v}_{i}t$ (superlubric movement). The other case is the decrements that disobey the linear relation ${u}_{c}={v}_{i}t$. These decrements mean that the CM of the 1DAC moves with a relatively small displacement although the 1DAC has a high initial velocity. In other words, a high initial velocity may not result in a large sliding distance even between atomically smooth interfaces. Such a finding is different from that for the classical sliding process. Therefore, to achieve superlubric sliding, a suitable rather than an arbitrary initial velocity should be selected.

**Table 1:** Initial positions of all the atoms in 1DAC.

*i*	1	2	3	4	5	6	7	8	9	10	11	12	13	14	15
*u*_*i*_/*a*_*s*_	0.25	1.25	2.25	3.25	4.25	5.25	6.25	7.25	8.25	9.25	10.25	11.25	12.25	13.25	14.25

**Figure 4:**
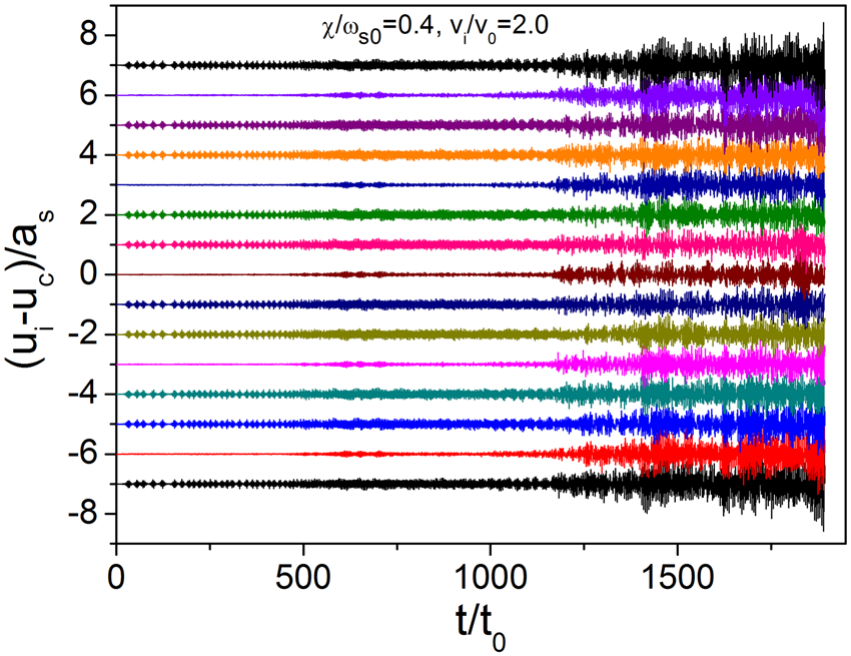
Displacement of atoms relative to CM as a function of time in consideration of substrate vibration caused by phonon excitation for *N* = 15, $k{a}_{{\rm{s}}}=0.15\pi$, and $\chi /{\omega }_{s0}=0.4$.

**Figure 5:**
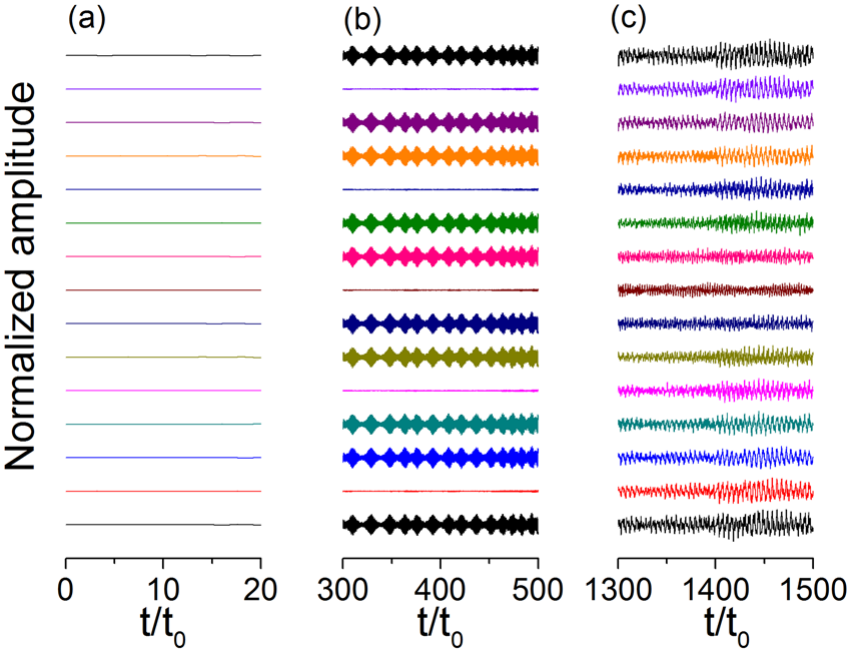
Normalized amplitude for the time span of (**a**) 0–20, (**b**) 300–500, and (**c**) 1300–1500.

To understand the physical mechanism of the decrements in Fig. 3, we examined the dynamic characteristics of the atomic chain in relation to the substrate lattice vibrations. Figure 4 presents the displacement of the atoms relative to CM as a function of time for $\chi /{\omega }_{{\rm{s0}}}=0.4$, ${v}_{i}/{v}_{0}=2.0$, and *N* = 15. The sliding process can be divided into three stages. Figure 4 provides an overview in the interval of $0 < t/{t}_{0} < 1893$, and Fig. 5 presents an enlarged view of the relative displacements of the different atoms for the three different intervals of $0 < t/{t}_{0} < 20$ (a), $300 < t/{t}_{0} < 500$ (b), and $1300 < t/{t}_{0} < 1500$ (c) corresponding to the three typical stages. In the initial stage shown in Fig. 5(a), the atoms vibrate with very small amplitudes. In the middle stage shown in Fig. 5(b), the second, fifth, eighth, eleventh, and fourteenth atoms vibrate with very small amplitudes, whereas the other atoms vibrate with large amplitudes. Comparison of Figs 5(b) and 2(j) shows that these amplitudes are consistent with the phonon mode of $\omega /{\omega }_{{\rm{s0}}}=1.0$, which means the corresponding phonon is excited. In the third stage, the vibration with a large amplitude is highly complicated, and the system shifts to an unstable state.

**Figure 6:**
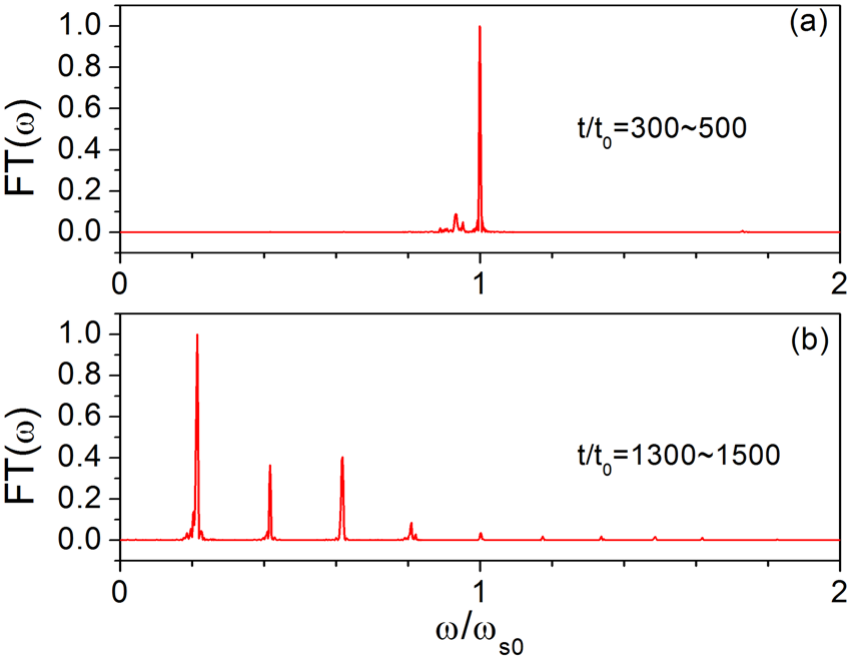
Resonant excitation peaks of (**a**) single phonon mode and (**b**) multi-phonon mode numerically obtained in the time range of 300–500 and 1300–1500, respectively.

To confirm phonon excitation, Fourier transform was conducted for the different time stages, as shown in Fig. 6. The peak frequency appears at $\omega /{\omega }_{{\rm{s0}}}=1.0$ for the time range of $300 < t/{t}_{0} < 500$ in Fig. 6(a). This peak frequency and the relative amplitude correspond to the phonon excitation of the $\omega /{\omega }_{{\rm{s0}}}=1.0$ mode and indicate that in this stage, the agreement between the eigenfrequency and the peak in the Fourier transform spectrum is good. In this stage, only the single phonon is excited. However, after Fourier transform for the time range of $1300 < t/{t}_{0} < 1500$, we observed several peaks, as shown in Fig. 6(b). The values of these resonance frequencies are in good agreement with the eigenfrequencies shown in Fig. 2. Compared with that in Fig. 6(a), the phonon excitation at $\omega /{\omega }_{s0}=1.0$ fades, and its energy transfers to the low-frequency phonon. This condition shows that excitation of multiple phonon modes occurs during the sliding process when time $t/{t}_{0}$ ranges from 1300–1500.

**Figure 7:**
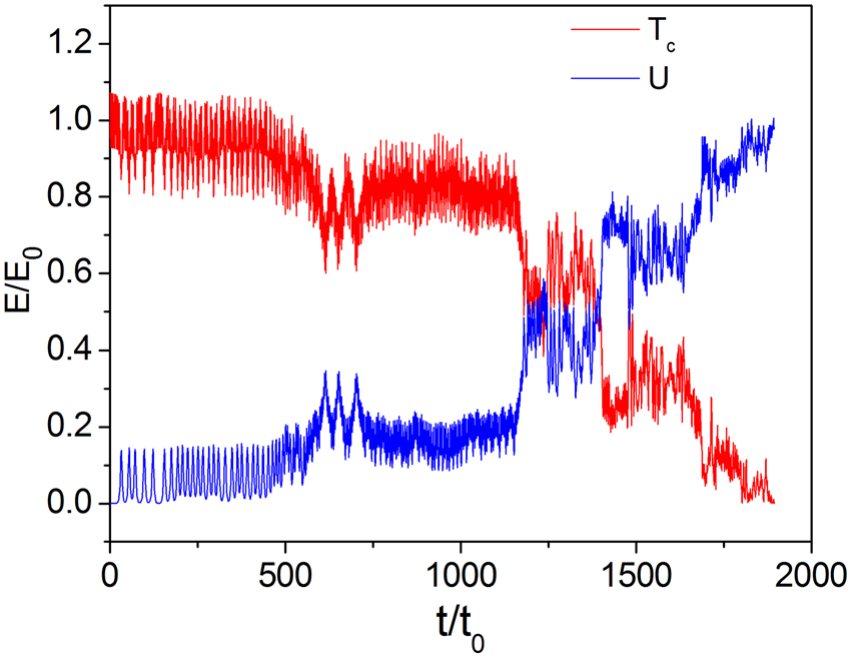
Evolution of kinetic energy ${T}_{c}$ of CM and internal energy *U* of the system for $N=15$, $k{a}_{{\rm{s}}}=0.15\pi$, and $\chi /{\omega }_{s0}=0.4$.

Energy evolution was also investigated. Figure 7 shows the time evolution of kinetic energy *T*_*c*_ and internal energy *U* for initial velocity ${v}_{i}/{v}_{0}=2.0$, where *E*_0_ is the initial total energy of the system. In the initial stage, kinetic energy of CM is stable, and the internal energy is almost constant because the relative displacement is small. This stage corresponds to a superlubric sliding process. In the next stage, the single-mode phonon begins to be excited, and kinetic and internal energies change each other and exhibit oscillation behaviors. In this stage, the kinetic energy is not significantly dissipated. After $t/{t}_{0}\approx 1160$, in the third stage, multi-phonon excitation leads to the decay of kinetic energy. The kinetic energy of CM is transformed into internal energy, and the amplitude of the atoms increases significantly. Potential energy is in fact a part of internal energy (the kinetic energy of atoms relative to CM plus the potential energy). The transformation of the kinetic energy of CM into internal energy is irreversible. Internal energy increases continuously, so the velocity of CM decreases continuously until the kinetic energy of CM disappears. This scenario explains the strong energy dissipation process by phonon excitation.

Notably, the vibration of atoms in the substrate enhances the phonon excitation. Owing to the vibrations of atoms near their equilibrium positions both in 1DAC and substrate, the possibility that two atoms in 1DAC fall into the interval between two atoms in the substrate or that two atoms in the substrate fall into the interval between two atoms in 1DAC increases. Such a configuration has high elastic potential, which can easily lead to phonon excitation. Hence, we considered the vibration of the substrate atoms. Compared with the substrate described by a fixed cosine-type periodic potential, the modified substrate potential that considers vibration is more suitable for describing the real process.

**Figure 8:**
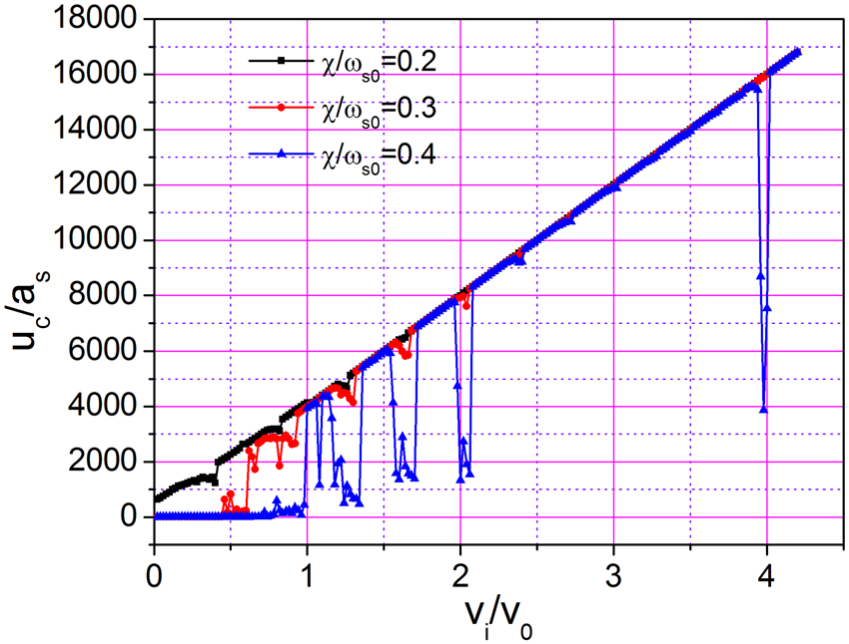
Largest displacement of CM as a function of initial velocity for *N* = 15, $k{a}_{{\rm{s}}}=0.15\pi$, and different $\chi /{\omega }_{s0}$ in the time range of 0–4000.

Next, we investigated the dependence of the sliding distance of CM on initial velocity for different coupling strengths $\chi /{\omega }_{s0}$. The result is shown in Fig. 8. When the coupling strength is low (black curves, $\chi /{\omega }_{s0}=0.2$), only a few drops appear in the displacement–velocity curve. The displacement of CM is proportional to initial velocity at nearly all velocities, indicating that superlubricity can be easily obtained for an arbitrary velocity with weak coupling. However, when the coupling strength is high, although most regions on the curves are still linear, a large number of drops appear. In particular, when coupling strength $\chi /{\omega }_{s0}$ is 0.4 (see blue curve), the 1DAC almost cannot move with low initial velocity. Furthermore, the 1DAC cannot move a long distance even with a large initial velocity of ${v}_{i}/{v}_{0}=4$ at $\chi /{\omega }_{s0}=0.4$. Such a phenomenon does not occur in our daily lives. The sudden decrease in displacement indicates that significant dissipation of kinetic energy occurs in the sliding process when the initial velocities are exactly at these values. For an atomically smooth interface, energy transfer can only occur between kinetic and potential energy. Given that the kinetic energy of 1DAC is transformed into internal energy that leads to phonon excitation, the 1DAC cannot move far. We conclude that the higher the coupling strength is, the more phonons are excited.

Because of the vibration of the substrate, 1DAC feels a nonperiodic rather than a strict periodic driving force. The break down of linear relationship between initial velocity and displacement is determined by the eigenfrequencies obtained from Eq. (10) and can be obtained only when the phonon mode is quantized. When the number of atom is infinite, the discrete phonon mode will be replaced by a continuous one. In the process of movement of 1DAC, when the phonon is excited, the linearity between the sliding distance and initial velocity will break down. As long as these phonon eigenfrequencies exist, the break down of the linearity can’t be avoided. However, we can’t determine the moment at which the phonon is excited, and it is difficult to obtain the analytic formula between 1DAC phonon eigenfrequencies and the initial sliding velocities when the linearity breaks down.

**Figure 9:**
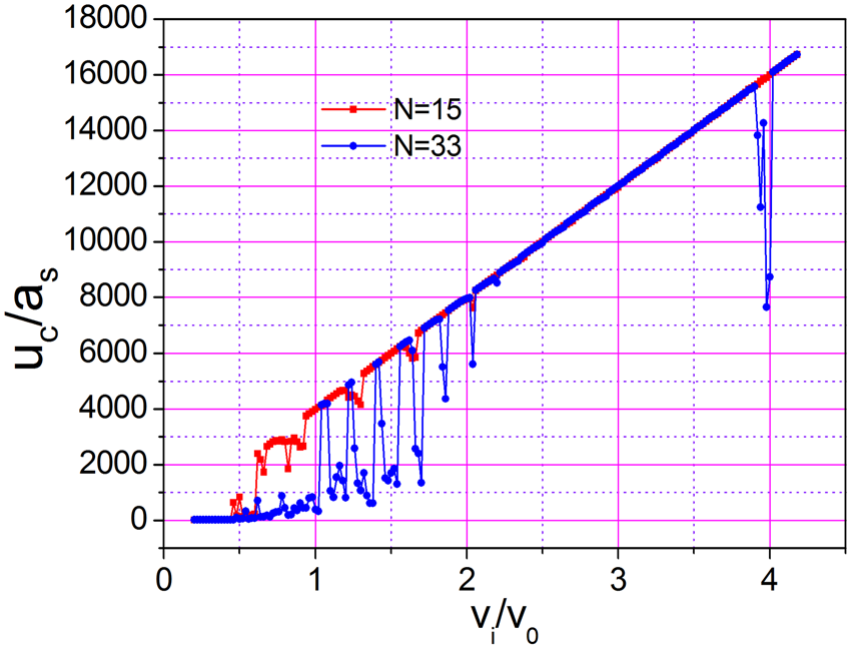
Largest displacement of CM as a function of initial velocity for $k{a}_{{\rm{s}}}=0.15\pi$, $\chi /{\omega }_{s0}=0.3$, and (**a**) *N* = 15, (**b**) *N* = 33 in the time range of 0–4000.

Lastly, we investigated the influence of the size of 1DAC on sliding movement. Figure 9 shows the dependence of the sliding distance of 1DAC with 33 atoms (blue curve) in contrast with that with 15 atoms (red curve). The coupling strengths, $\chi /{\omega }_{s0}$, are both 0.3. The 1DAC with 33 atoms and that with 15 atoms exhibited a similar behavior. However, compared with the red curve, the blue curve contains more drops, indicating that the 1DAC with 33 atoms has more eigenfrequencies that correspond to phonon excitation. As the number of atoms decreases, the number of excited modes decreases, and the possibility of energy dissipation decreases. Therefore, we conclude that a critical size exists on the atomic level, below which no dissipation occurs when the 1DAC is sliding on the substrate. Such a result is similar to that of Sokoloff^9^.

The use of a modified substrate potential is advantageous. In fact, the motion of atoms on the substrate cannot be avoided, thus leading to a variation in the potential over time. Consequently, a pair of neighbor atoms in the 1DAC may be trapped between two neighbor atoms on the substrate, thus increasing the potential energy. Such a process easily causes phonon excitation and leads to the dissipation of kinetic energy of 1DAC.

In addition, for a finite two-dimensional graphene, we can also obtain a discrete dispersion relation of phonon similar with that of 1DAC. In the sliding process of the graphene, the phonon has the possibility to be excited, so it might be expected that there are still drops in the curve of the sliding distance and initial velocity.

## Conclusion

The dynamic behaviors of a 1DAC sliding on a substrate were studied with a modified Frenkel–Kontorova model. The dependence of the sliding distance of the 1DAC on initial velocity and energy dissipation was also investigated in detail. The sliding distance of 1DAC is closely related to its initial velocity. Although superlubricity is likely to be achieved in an atomically smooth interface at an arbitrary initial velocity, several special velocities still exist; at these velocities, the kinetic energy of 1DAC dissipates rapidly because of phonon excitation. The physical mechanism of the energy dissipation of the sliding 1DAC was also analyzed. The sliding process can be divided into three stages. In the first stage, the 1DAC experiences a superlubric process because of the transformation of the contact interface from commensurate to incommensurate. With the motion of the 1DAC, the atoms exhibit harmonic oscillation because of single-phonon excitation in the second stage. In the third stage, multi-phonon excitation leads to the transfer of the kinetic energy of the 1DAC to the internal energy of the total system. Furthermore, the relationship between sliding distance and initial velocity depends on the coupling strength and size of the 1DAC. High coupling strength and large atoms in the 1DAC lead to high possibility of energy dissipation. Our findings not only reveal the energy transformation in the sliding process but are also helpful in achieving superlubricity.
